# Value of Laboratory Tests in Employer-Sponsored Health Risk Assessments for Newly Identifying Health Conditions: Analysis of 52,270 Participants

**DOI:** 10.1371/journal.pone.0028201

**Published:** 2011-12-07

**Authors:** Harvey W. Kaufman, Fred R. Williams, Mouneer A. Odeh

**Affiliations:** Quest Diagnostics, Madison, New Jersey, United States of America; Yale University School of Medicine, United States of America

## Abstract

**Background:**

Employer-sponsored health risk assessments (HRA) may include laboratory tests to provide evidence of disease and disease risks for common medical conditions. We evaluated the ability of HRA-laboratory testing to provide new disease-risk information to participants.

**Methodology/Principal Findings:**

We performed a cross-sectional analysis of HRA-laboratory results for participating adult employees and their eligible spouses or their domestic partners, focusing on three common health conditions: hyperlipidemia, diabetes mellitus, and chronic kidney disease. HRA with laboratory results of 52,270 first-time participants were analyzed. Nearly all participants had access to health insurance coverage. Twenty-four percent (12,392) self-reported one or more of these medical conditions: 21.1% (11,017) self-identified as having hyperlipidemia, 4.7% (2,479) self-identified as having diabetes, and 0.7% (352) self-identified as having chronic kidney disease. Overall, 36% (n = 18,540) of participants had laboratory evidence of at least one medical condition newly identified: 30.7% (16,032) had laboratory evidence of hyperlipidemia identified, 1.9% (984) had laboratory evidence of diabetes identified, and 5.5% (2,866) had laboratory evidence of chronic kidney disease identified. Of all participants with evidence of hyperlipidemia 59% (16,030 of 27,047), were newly identified through the HRA. Among those with evidence of diabetes 28% (984 of 3,463) were newly identified. The highest rate of newly identified disease risk was for chronic kidney disease: 89% (2,866 of 3,218) of participants with evidence of this condition had not self-reported it. Men (39%) were more likely than women (33%) to have at least one newly identified condition (p<0.0001). Among men, lower levels of educational achievement were associated with modestly higher rates of newly identified disease risk (p<0.0001); the association with educational achievement among women was unclear. Even among the youngest age range (20 to 29 year olds), nearly 1 in 4 participants (24%) had a newly identified risk for disease.

**Conclusions/Significance:**

These results support the important role of employer-sponsored laboratory testing as an integral element of HRA for identifying evidence of previously undiagnosed common medical conditions in individuals of all working age ranges, regardless of educational level and gender.

## Introduction

Employer-sponsored health risk assessments (HRA), a cornerstone of wellness programs, are used to engage adults in taking responsibility for their own health, provide feedback that will lead to behavioral modifications that reduce disease risks, raise productivity [1], [2], and control healthcare expenses [3]. Simply participating in HRA appears to have benefit [4], [5]. HRA are now being offered by more than 75% of mid- to large-sized companies [6].

HRA involve a health and lifestyle questionnaire and may include biometric data (such as weight, body mass index (BMI), waist circumference, and blood pressure) and laboratory test results. The inclusion and selection of laboratory tests vary by program. Self-reported results, even for widely known disease risk factors such as total cholesterol, are often inaccurate [7]. When measured laboratory tests are included in HRA, they are typically limited to total cholesterol, lipid panel (total cholesterol, HDL cholesterol, calculated LDL cholesterol, and triglycerides) and fasting glucose. It is less typical to include tests for other common conditions, such as chronic kidney disease.

This study explores the utility of employer-sponsored laboratory testing in revealing for new participants evidence of three common medical conditions: hyperlipidemia, diabetes mellitus, and chronic kidney disease. These medical conditions were selected because of their high prevalence in the United States population and the benefit of effective intervention associated with early detection. Cardiovascular disease is listed on more than half of all death certificates [8], and diabetes [9] and chronic kidney disease [10] are each estimated to affect approximately 26 million Americans.

Current guidelines for “normal-risk” individuals suggest that screening begin at age 40 years for cardiovascular disease [11], 45 years for diabetes [12], and 60 years for chronic kidney disease [13]. However, these diseases are growing in prevalence, even in younger adults, and are often not diagnosed in a timely fashion [14]–[15], [16], [17], [18]. Early detection and treatment can delay or arrest disease progression, and may help to avoid co-morbid complications. The importance of screening for chronic kidney disease in particular is often overlooked, increasing the likelihood for associated kidney failure and cardiovascular events [13], [19].

The role and value of laboratory tests within employer-sponsored HRA have not been well investigated. Among these limited studies, none appears to have evaluated how often common chronic diseases are *newly* identified among such participants. We explore the frequency of results that are consistent with increased disease risk and compare this with self-reported information. This study uniquely focuses on the impact of age, gender, and education level on the new identification of common health conditions. Additionally, this study is the first to assess the role of laboratory testing for chronic kidney disease as part of an employer-sponsored HRA.

## Materials and Methods

### Study Population

This was a cross-sectional study of 52,270 first-time participants ages 20 to 64 years using the Quest Diagnostics Blueprint for Wellness® HRA. The program was sponsored by 15 employers representing diverse industries between 2003 and 2010 with participants from across the United States. To isolate the extent to which laboratory testing provides new and medically relevant health risk information, the study was limited to first-time participants. Participants 65 years and older were excluded from this study because of the relatively small number. The analysis in this study was found to be exempt by the Western Institutional Review Board for the protection of human subject research.

Participants included adult employees and their eligible spouses or their domestic partners. All of the employers offered healthcare insurance coverage to their eligible employees and covered family members.

### Evidence of Medical Conditions: Laboratory-Based and Self-reported Awareness

Three common measurements of hyperlipidemia were used: total cholesterol above 5.15 millimoles per liter (199 milligrams per deciliter) [11], low density lipoprotein (LDL) cholesterol above 3.35 millimoles per liter (129 milligrams per deciliter), and total cholesterol to high density lipoprotein (HDL) cholesterol ratio above 5.0. Hyperlipidemia was identified if one or more of these criteria were met. A diagnosis of diabetes mellitus can be based on fasting glucose levels or hemoglobin A1c levels. For this study, an elevated fasting glucose level (greater than 6.90 millimoles per liter (125 milligrams per deciliter)) was considered evidence of diabetes. Kidney function was assessed with the estimated glomerular filtration rate (eGFR), a calculation based on serum creatinine, age, gender, and ethnicity (whether one is identified as African American or non-African American). eGFR values below 60 mL/min/1.73 m^2^ are indicative of chronic kidney disease stages 3 to 5 [13]. When ethnicity was not reported, eGFR was based on the non-African American calculation. In a small percentage of participants (estimated to be fewer than 75 out of 3,218 participants with eGFR results below the threshold), this could overstate the identification of new chronic kidney disease risk. This estimate is based on taking all eGFR results between 50 and 59 ml/min/1.73 m^2^, inclusive, for whom ethnicity was not identified (n = 527) and multiplying by the percent of African-Americans identified in the study population (14.0%). This provided an estimate of the number of unidentified African Americans who if they were so identified may have had a higher eGFR based on the MDRD Study equation adjustment factor of 1.21 that applies only to African Americans.

As part of the HRA survey, participants were asked to report whether they had been informed by a physician that they had any of the medical conditions included in this study. For these conditions, a comparison of self-reported disease awareness with laboratory-based criteria provides an indication of new medical risk information for each participant. Thus, a medical condition was considered to be newly identified if it was not self-reported but the relevant laboratory result(s) exceeded the threshold value. For example, if a participant reported that he or she had not been diagnosed with diabetes and the fasting glucose results showed evidence of diabetes (fasting glucose >125 mg/dL), we classified the participant as having newly identified diabetes. However, it is important to note that laboratory results comprise only part of the diagnostic pathway; further medical evaluation is required for diagnosis. Furthermore, this methodology only provides a snapshot of health risk and does not include health risks that may be reflected in longer-term trends. We used the term “newly identified” because the medical risk is not “diagnosed” until confirmed usually with testing on a second specimen and after excluding other causes of the laboratory finding. Also, we suspect that some participants may have exceeded the defined criteria earlier but were not informed by their physicians. Thus, we view the HRA serving to identify rather than diagnose new disease risks.

### Statistical Analysis

Table 1 lists the measures used to assess prevalence of disease risk and the ability of employer-sponsored HRA with laboratory testing to identify previously unrecognized medical conditions.

**Table 1:** Measurements Used to Characterize Disease Prevalence and the Rate of Newly Identified Disease Risk.

Metric	Description
**Total Disease Prevalence**	Proportion of first time participants who self-reported medical condition or were newly identified disease risk as a result of employer-sponsored laboratory testing.
**Rate of Newly Identified Disease Risk**	Proportion of the total disease population who found out about their health risks from employer-sponsored laboratory testing.

Total disease risk prevalence was defined as the proportion of participants who self-reported medical condition or had identified disease risk as a result of employer-sponsored laboratory testing. Unlike other studies that define prevalence on either self-identification of disease or laboratory testing, this approach provides a broader definition of disease prevalence. This enables us to isolate the contribution of employer-sponsored laboratory testing in the identification of previously unrecognized disease risk.

The rate of newly identified risk was defined as the proportion of the total risk-identified population who found out about their health risks from employer-sponsored laboratory testing.

For each disease, risk prevalence and rate of newly identified risk are explored by age range, gender, and education level. To provide greater insight, we characterized the distribution of participants by the number of disease risks identified. The number of disease risk identifications for any individual participant ranged from zero to three (one newly identified risk for each of the three medical conditions analyzed).

Statistical significance is evaluated using Pearson's chi-squared test statistic based on two-sample tests of proportion, performed using R 2.12.2 (prop.test). Statistical significance for p-value <0.0001 was reported. Gender comparisons were evaluated with men as the baseline distribution; age comparisons were evaluated with 50 to 64 year old participants as the baseline distribution; and the distribution of participants with high school or less education was used as the baseline for evaluation of educational impact.

## Results

### Study Population

Table 2 summarizes the age and gender distributions of the study population. The average ages of men and women were 43.4 and 41.7 years, respectively. Women comprised 62.5% of the study population. Relatively more women than men were represented in the youngest age range (20 to 29 years) and relatively more men than women were represented in the oldest age range (50 to 64 years). Compared to the overall United States population [20], the study population had a relatively larger proportion of individuals in the 30 to 49-year age range and a relatively smaller proportion in other age ranges, particularly the youngest age group.

**Table 2:** Description of Study Population.

	Percent of Study Population(n = 52,270)	Percent AmongMen(n = 19,593)	Percent AmongWomen(n = 32,677)
***Age Range (years)***			
20–29	15.3	12.5	16.9
30–39	25.5	24.8	26.0
40–49	30.0	30.4	29.8
50–64	29.2	32.3	27.3
Average Age	42.3 years	43.4 years	41.7 years
***Education*** †			
High School (or less)	16.2	17.5	15.4
Some College	33.0	28.9	35.4
Bachelor's Degree	35.7	36.5	35.3
Graduate Degree	15.1	17.2	13.9
***Ethnicity*** ‡			
Caucasian	56.0	58.5	54.5
Asian	16.0	17.7	15.0
African American	14.0	9.4	16.7
Hispanic	10.5	11.0	10.2
American Indian	0.4	0.4	0.4
Other	3.1	3.0	3.2
***U.S. Region*** *			
New England	3.7	3.6	3.7
Mid-Atlantic	17.5	17.9	17.2
East North Central	9.1	9.0	9.2
West North Central	7.5	7.7	7.4
South Atlantic	26.9	22.6	29.3
East South Central	2.1	2.3	1.9
West South Central	13.3	16.4	11.6
Mountain	4.7	4.4	4.9
Pacific	15.2	16.1	14.6

† Educational attainment was not reported for 0.8% of participants. “Some College” educational category includes vocational training and associate's degree.

‡ Ethnicity was not reported for 22.9% of participants. “Other” ethnicity category includes responses for “other” and “multi-ethnic”.

* State of residence was not reported for 10.2% of study population participants.

Participants came from across the United States, with over-representation relative to the 2009 United States population [21] in the Mid- and South-Atlantic census regions, and relative under-representation in the East North Central and East South Central census regions (state of residence was not reported for 10.2% of study population participants). The study population was racially diverse, with a higher percentage of Asians relative to the similarly-aged adult United States population. Caucasians and Hispanics were underrepresented relative to the general population (ethnicity was not reported for 22.9% of participants).

The study population had a higher representation of high educational achievement than the general population (educational attainment was not reported for 0.8% of participants). Overall, 50.8% of the study population had a bachelor or graduate degree. This compares to only 31.4% for the general United States population aged 25 years or older.

### Prevalence of disease by Self-Identification

Twenty-four percent (12,392) who self-reported one or more of these medical conditions: 21.1% (11,017 self-identified as having hyperlipidemia, 4.7% (2,479) self-identified as having diabetes, and 0.7% (352) self-identified as having chronic kidney disease Table 3). Men were more likely than women to have each of the three medical conditions. The prevalence for each of the three medical conditions increased by age range: among the 50 to 64 year old participants, hyperlipidemia was self-identified in 36.3%, diabetes in 11.8%, and chronic kidney disease in 12.1%. There was no clear relationship between educational achievement and self-identification of hyperlipidemia or chronic kidney disease whereas there was an inverse relationship between educational achievement and the self-reported rate of diabetes.

**Table 3:** Disease Prevalence and Percent Newly Identified by Gender, Age Range, and Educational Level.

		Hyperlipidemia#				Diabetes†				Chronic Kidney Disease‡			
	Number of Participants	Overall Prevalence (Percent)	Self-Reported Prevalence(Percent)	Laboratory Identified Incidence (Percent)	Rate of Laboratory Identification (Percent)	Overall Prevalence (Percent)	Self-Reported Prevalence(Percent)	Laboratory Identified Incidence (Percent)	Rate of Laboratory Identification (Percent)	Overall Prevalence (Percent)	Self-Reported Prevalence(Percent)	Laboratory Identified Incidence (Percent)	Rate of Laboratory Identification (Percent)
**Study Population**	52,270	51.7	21.1	30.7	59.3	6.6	4.7	1.9	28.4	6.2	0.7	5.5	89.1
***Gender***													
Men	19,593	60.3	25.6	34.7	57.5	8.0	5.6	2.4	30.5	5.7	0.7	4.9	87
Women	32,677	46.6^a^	18.4	28.2	60.6^a^	5.8^a^	4.2	1.5	26.7^a^	6.4^a^	0.6	5.8	90.2^a^
***Age Range***													
20–29 years	7,979	29.2^b^	5.7	23.5	80.6^b^	1.7^b^	1.1	0.7	38.7^b^	1.0^b^	0.4	0.6	61.4^b^
30–39 years	13,348	42.5^b^	12.8	29.7	69.9^b^	3.5^b^	2.4	1.1	31.7^b^	2.7^b^	0.4	2.2	83.1^b^
40–49 years	15,688	54.5^b^	21.1	33.3	61.2^b^	6.8^b^	4.5	2.3	33.2^b^	5.9^b^	0.6	5.3	89.3^b^
50–64 years	15,255	68.8	36.3	32.5	47.2	11.8	8.9	2.8	23.9	12.1	1.0	11.1	91.4
***Education***													
High school or less	8,386	56.6	23.9	32.7	57.8	9.2	6.6	2.6	28.5	6.0	0.6	5.4	90.3
Some college or vocational training	17,091	51.4^c^	21.4	30.1	58.4	7.5^c^	5.4	2.1	27.4^c^	5.7	0.7	5.0	87.1^c^
Bachelor's degree	18,527	49.7^c^	19.6	30.1	60.6^c^	5.3^c^	3.7	1.6	30.1^c^	5.9	0.6	5.3	89.8
Graduate degree	7,833	51.8^c^	20.8	31.0	59.8^c^	5.0^c^	3.6	1.4	27.4^c^	7.7^c^	0.8	6.9	89.9

# Risk of hyperlipidemia is indicated if one of three common laboratory measurements of hyperlipidemia exceeded clinical thresholds: total cholesterol above 5.15 millimoles per liter (199 milligrams per deciliter) [36], low density lipoprotein (LDL) cholesterol above 3.35 millimoles per liter (129 milligrams per deciliter), and total cholesterol to high density lipoprotein (HDL) cholesterol ratio above 5.0.

† Risk of diabetes mellitus is indicated for elevated fasting glucose level (greater than 6.90 millimoles per liter (or 125 milligrams per deciliter)).

‡ Kidney function was assessed using estimated glomerular filtration rate (eGFR), based on serum creatinine, age, gender, and ethnicity (whether one is identified as African American or non-African American). eGFR values below 60 mL/min/1.73 m^2^ are indicative of chronic kidney disease stages 3 to 5.

a p-value<0.0001 for comparison with men.

b p-value<0.0001 for comparison with 50–64 year olds.

c p-value<0.0001 for comparison with high school or less.

### Incidence of Newly Identified Disease Risk

More than a third (35.5%) of participants had laboratory evidence of one or more disease risk identified through HRA-laboratory testing (Table 4). Of these, 92.9% had laboratory evidence of one, 7.0% had evidence of two, and 0.1% had evidence of all three conditions identified. The rate of newly identified disease risk increased progressively with age, ranging from 24.4% among 20 to 29 year olds to 41.7% among 50 to 64 year olds. Among men, lower levels of educational achievement were associated with modestly higher rates of newly identified disease risk, ranging from 41.8% for men with high school or less to 37.0% for men with graduate degrees (p-value<0.0001). There was no clear relationship between educational achievement and newly identified disease risk among women. Additionally, we observed no clear trend in the rate of disease risk identification over the study period.

**Table 4:** Percent of Participants with One or More Newly Identified Disease Risks.

	Total	Men	Women
**Study Population**	35.5	39.4	33.1^a^
***Age Range, years***			
20–29	24.4^b^	31.1^b^	21.4^a^^,^ b
30–39	31.8^b^	41.5^b^	26.3^a^^,^ b
40–49	38.1^b^	42.8^b^	35.2^a^^,^ b
50–64	41.7	37.7	44.6^a^
***Education***			
High school or less	37.9	41.8	35.3^a^
Some college or vocational training	34.6^c^	40.0^c^	32.0^a^^,^ c
Bachelor's degree	34.8^c^	38.9^c^	32.3^a^^,^ c
Graduate degree	36.3^c^	37.0^c^	35.7^a^

a p-value<0.0001 for comparison with men.

b p-value<0.0001 for comparison with 50–64 year olds.

c p-value<0.0001 for comparison with high school or less.

### Hyperlipidemia

Overall, more than half of the participants (51.7%) either self-reported hyperlipidemia or had laboratory evidence of hyperlipemia newly identified through the HRA (Table 4). The prevalence of hyperlipidemia risk was significantly higher among men (60.3%) than women (46.6%) (p-value<0.0001). While age is a significant risk factor, we found that the risk for hyperlipidemia began at an early age, affecting 29.2% of the youngest age group and increased steadily in older groups.

Of participants with self-reported or laboratory evidence of hyperlipidemia, the majority (59.3%) were newly identified through the HRA program. The rate of newly identified laboratory evidence of disease was high for both men (57.5%) and women (60.6%), and was especially high among the youngest age range (80.6%). Even among the oldest age group, nearly half (47.2%) of those with evidence of hyperlipidemia were newly identified through the HRA. No clear trend was observed between educational attainment and newly identified risk of hyperlipidemia.

### Diabetes Mellitus

The total diabetes risk prevalence was 6.6% in our study population (Table 4). This included participants who self-reported a medical condition or newly identified their health risk through the HRA. The diabetes risk prevalence was significantly higher among men (8.0%) than women (5.8%) (p-value<0.0001). Risk for diabetes began at an early age, affecting 1.7% of adults aged 20 to 29 and 3.5% of adults aged 30 to 39 years. Among 50 to 64 year olds, the risk of diabetes was 11.8%. There was an inverse relationship between advancing educational attainment and diabetes risk prevalence: diabetes risk was found in 9.2% of adults with high school education or less and 5.0% of those adults with graduate degrees.

Among participants with self-reported or laboratory evidence of diabetes, 28.4% were newly identified through this program. Newly identified disease risk was slightly higher among men (30.5%) than women (26.7%) (p-value<0.0001). Nearly 2 in 5 (38.7%) participants with diabetes risk in the youngest age range (20 to 29) were newly identified; nearly 1 in 4 (23.9%) participants between 50 and 64 years old were newly identified. No trend was observed between educational attainment and risk of newly identified diabetes risk.

### Chronic Kidney Disease

The total chronic kidney disease risk prevalence was 6.2% in our study population (Table 4). This included participants who self-reported or newly identified their chronic kidney disease risk through the HRA. The chronic kidney disease risk prevalence was slightly lower among men (5.7%) than women (6.4%) (p-value<0.0001). Risk for chronic kidney disease was identified beginning at an early age, affecting 1.0% of adults aged 20 to 29 and 2.7% of adults aged 30 to 39 years. Among 50 to 64 year olds, the risk of chronic kidney disease was 12.1%. As noted in Table 4, the prevalence of total disease risk was similar for chronic kidney disease and diabetes.

Among participants with self-reported or laboratory evidence of chronic kidney disease, 89.1% were newly identified through this program. The rates of newly identified disease risk were slightly lower among men (87.0%) than women (90.2%) (p-value<0.0001), but age had a marked effect on the likelihood of a disease being newly identified. No trend was observed between educational attainment and risk of newly identified chronic kidney disease.

## Discussion

More than one in three study participants had laboratory evidence of at least one common medical condition newly identified. The results of this study support the use of laboratory testing along with HRA questionnaires to identify previously unrecognized disease risk in individuals of all working age ranges, regardless of educational level and gender.

Ideally adults with healthcare insurance would avail themselves of medical services that are typically covered by their health plans, including routine physical examinations and laboratory tests. Yet, many adults do not seek preventive medical care in the absence of symptoms [22]. In one poll of men, 36% indicated they would go to the doctor only if “extremely sick” [23]. HRA with a laboratory component can address this shortcoming by uncovering disease risk factors and driving participants to seek medical care when risks are identified. Recognizing this, many employers increasingly offer HRA with laboratory tests.

### Large Number of Participants with Newly Identified Risk

The percent of participants with newly identified disease risk (35.5%) was significantly higher than the percent of participants who self-identified (23.7%) as having one or more of these three conditions. This high rate of newly identified risk suggests that our current healthcare system fails to identify common disease risk factors for a large number of working-age people, even for those with access to quality healthcare. Our study also suggests that without employer-sponsored laboratory testing, more than 1 in 3 working-age adults may have unidentified disease(s). When left untreated these conditions can progress to more advanced stages with irreversible harm and needless expense. If we extend this observation to the United States population aged 20 to 64 years, 67 million Americans have undiagnosed laboratory markers for hyperlipidemia, diabetes, and/or chronic kidney disease. Our findings are consistent with broader population studies that include non-working adults of all ages [15], [24], [25], and further accentuate the importance of including laboratory testing in employer-sponsored HRA.

### Importance of Testing for Chronic Kidney Disease

The high prevalence of diabetes (25.8 million in the United States) is well recognized. Although chronic kidney disease has a similar prevalence (26.3 million) [26] awareness of this condition is low even among those affected. In our study, 89.1% of participants with self-reported or laboratory evidence of chronic kidney disease were unaware of their condition. This compares to 28.4% for diabetes and 59.3% for hyperlipidemia. Early detection and treatment can slow or halt the progression of chronic kidney disease and associated co-morbidities [27], including cardiovascular disease and diabetes. The findings of this study complement other research supporting the inclusion of chronic kidney disease testing in employer-sponsored HRA [28].

### Benefits of Testing Are Widespread

Our study found that the benefits of employer-sponsored laboratory testing are widespread, with high rates of disease risk identification for both men and women, and across all age ranges and educational achievement.

While the rates of disease risk identification were high for all populations, some groups were impacted more significantly than others. Men under the age of 50 years had a higher rate of newly identified risk than women. This may be due, in part, to lower outpatient physician visit rates among men relative to women [29]. Interestingly, the pattern of newly identified disease risk reverses among participants 50 to 64 years of age, with women having a higher rate of newly identified disease risk than men. While the reason for this reversal is unclear, it is possible that men in their 50 s are increasingly aware and responsive to guidelines suggesting population screening for a variety of medical conditions, including general guidelines for colorectal cancer screening and discussion of prostate cancer screening beginning at age 50 years [30].

Professional guidelines typically recommend testing be initiated in middle-aged and older adults who are not otherwise at high disease risk [12], [31]. Younger people are less likely to have a personal physician and to seek preventative medical care. Studies have shown that younger adults are heavier [32] and more sedentary than ever, and therefore exhibit many more risk factors than earlier generations for the three medical conditions included in this study. Our study suggests that these three conditions are more common among younger aged adults than is generally recognized. For participants with evidence of hyperlipidemia or diabetes, our study found that younger participants were less likely to be aware of their condition. Even though the rate of newly identified chronic kidney disease increases with age, more than 3 out of 5 of the youngest participants with chronic kidney disease were newly identified. Our study demonstrates that employer-sponsored HRA with laboratory testing reveals important health risk information even for younger ages. This supports the value of offering broad-based population screening across all ages.

Many studies from around the world support the relationship between health literacy and better health outcomes [33]–[34], [35], [36]. Although health literacy increases with educational attainment, it still remains below proficient for two-thirds of graduate degree recipients [37]. Our study suggests that educational achievement provides little benefit in the early identification of these three common diseases.

### Study Limitations

#### Cross-Sectional Population

This study reflects the specific health awareness and promotion activities performed by 15 employers. Experience of other employers may differ. The study population differs from the general working-age population in being more highly educated, over-representing women, and somewhat skewed based on geography and ethnicity. Contrary to prevalent beliefs, these factors had no or minimal impact on our findings suggesting that selection-bias would likewise have had no or minimal influence on our results. While these differences could limit the applicability of these results to the broader working-age population, our findings on disease risk prevalence are broadly consistent with large population studies [16], [24], [25].

The specific characteristics of incentive programs offered by the 15 employers may contribute to attracting different participants with different disease profiles. Further, employees already under medical care or in the other extreme, healthy employees, may have chosen not participate. Participants may have participated in other HRA prior to participation in Quest Diagnostics Blueprint for Wellness through their current or previous employers.

#### Accuracy of Self-Reported Disease Awareness

Preexisting knowledge of hyperlipidemia, diabetes, or chronic kidney disease is based on self-reported data. Differences in how terminology is understood may influence reported pre-existing awareness. Subjective factors regarding self-perception and denial may have adversely influenced how the respondents self-reported.

#### Disease Confirmation

Our disease classification is based on laboratory testing from a single blood collection. Although the diagnostic thresholds used are consistent with professional guidelines, a medical diagnosis requires confirmation of test results and syntheses with other clinical findings. Due to biological and analytical variation, it is commonly accepted clinical practice that for these three medical conditions individuals whose laboratory test results exceed diagnostic criteria be retested. Thus, the single observations in this study may over-estimate undiagnosed chronic medical conditions. For example, many factors influence measurement of fasting glucose [38]. An NHANES III study examining the reproducibility of fasting glucose testing in adults newly identified with diabetes showed that 70.4% had confirmation of their results [39]. Although confirmatory testing is appropriate on an individual basis, our methodology is consistent with other population-based studies, such as NHANES and the Framingham Heart Study, that use laboratory test results from a single snapshot in time.

### Future Directions

This study focuses on newly detected disease. Additional research is needed to assess the role of HRA in disease prevention. Furthermore, studies that track laboratory results of HRA participants over time are needed to assess and improve management of chronic diseases. Finally, the ability of HRA participation to modify behaviors (such as diet, exercise, smoking, and use of preventive services) needs to be investigated, particularly their impact on disease risks measured by laboratory tests.

Specific topics for future research include: 1) whether individuals with newly identified disease risk seek medical care; 2) the optimal frequency for employer-sponsored HRA with laboratory testing; and 3) the appropriate use of personalized testing based on demographic factors (e.g., age, gender, and/or education level), family history, and other biometrics such as BMI and blood pressure.

### Summary

In summary, our findings show that, for a large proportion of working-age adults, healthcare access alone does not guarantee detection of risk factors for common chronic health conditions. The availability of HRA with laboratory tests serves an important role in addressing this shortcoming. By identifying such opportunities early, employer-sponsored laboratory testing may slow or prevent the progression of common medical conditions. This has clear benefit to employees and their spouses and their domestic partners, regardless of age, gender, and educational achievement. Similarly, employers who bear much of the financial costs of poor disease management may benefit from early detection and treatment that can help to avert healthcare costs associated with advanced disease.
